# Microbial Recycling of Polylactic Acid Food Packaging Waste into Carboxylates via Hydrolysis and Mixed-Culture Fermentation

**DOI:** 10.3390/microorganisms11082103

**Published:** 2023-08-18

**Authors:** David P. B. T. B. Strik, Brian Heusschen

**Affiliations:** Environmental Technology, Wageningen University & Research, 6708 WG Wageningen, The Netherlands

**Keywords:** bioplastic, PLA, pretreatment, hydrolysis, fermentation, carboxylates, mixed culture, chain elongation, microbial recycling

## Abstract

To establish a circular economy, waste streams should be used as a resource to produce valuable products. Biodegradable plastic waste represents a potential feedstock to be microbially recycled via a carboxylate platform. Bioplastics such as polylactic acid food packaging waste (PLA-FPW) are theoretically suitable feedstocks for producing carboxylates. Once feasible, carboxylates such as acetate, n-butyrate, or n-caproate can be used for various applications like lubricants or building blocks for making new bioplastics. In this study, pieces of industrial compostable PLA-FPW material (at 30 or 60 g/L) were added to a watery medium with microbial growth nutrients. This broth was exposed to 70 °C for a pretreatment process to support the hydrolysis of PLA into lactic acid at a maximum rate of 3.0 g/L×d. After 21 days, the broths of the hydrolysis experiments were centrifugated and a part of the supernatant was extracted and prepared for anaerobic fermentation. The mixed microbial culture, originating from a food waste fermentation bioprocess, successfully fermented the hydrolyzed PLA into a spectrum of new C2-C6 multi-carbon carboxylates. n-butyrate was the major product for all fermentations and, on average, 6.5 g/L n-butyrate was obtained from 60 g/L PLA-FPW materials. The wide array of products were likely due to various microbial processes, including lactate conversion into acetate and propionate, as well as lactate-based chain elongation to produce medium-chain carboxylates. The fermentation process did not require pH control. Overall, we showed a proof-of-concept in using real bioplastic waste as feedstock to produce valuable C2-C6 carboxylates via microbial recycling.

## 1. Introduction

Plastic circularity and plastic pollution prevention have high priorities on political agendas [1,2]. A shift towards biobased biodegradable plastics which are effectively recycled could contribute to these goals [3]. Biodegradable plastics (BDPs) have intrinsic biodegradability properties which make them attractive to recycle via microbiological means. Currently, there is no highly effective practice for recycling biodegradable plastic products.

Presently, the main polymers produced for BDP-based products are polylactic acid (PLA), starch blends, polybutylene adipate terephthalate (PBAT), and polyhydroxyalkanoates (PHA) [4]. The actual biodegradation of various BDP polymers and the products made from them depend on the actual environmental conditions [5]. In certain areas, certified BDPs (like tea bags and coffee pads) are allowed to be disposed with the organic fraction of municipal organic waste (OF-MSW) [6]. Collection of BDP materials can stimulate the recovery of the tea, coffee, or food residues disposed with the organic waste streams to produce compost and/or biogas via anaerobic digestion [7].

Anaerobic digestion (AD) can degrade various BDPs using mixed-microbial cultures, allowing the bioconversion of complex waste materials like the OF-MSW or concentrated wastewaters. It is estimated is that there are more than 25,000 industrial-scale digesters [8]. The anaerobic digestion process typically includes four bioprocess steps: hydrolysis, acidogenesis, acetogenesis, and methanogenesis. Once the methanogenesis step is inhi-bited, carboxylates (especially short-chain fatty acids like acetate, propionate, and n-butyrate) accumulate as end products [9]. Once extra substrates, like ethanol or lactate, are added, the microbial chain elongation bioprocesses are induced, leading to the formation of medium-chain carboxylates like n-caproate and n-heptanoate [10,11]. Production of these carboxylates is part of the envisioned ‘carboxylate platform’ or ‘VFA (volatile fatty acid) platform’ [12,13]. Recently, several industries are operating or starting up commercial-scale production of such carboxylates to serve various applications [14,15,16].

Biodegradable plastics could become a resource not only to produce biogas, but to produce carboxylates as well. This could, in theory, be used to reproduce bioplastics like PHA [17]. Several studies have promoted the idea to use BDPs as feedstock for microbial recycling processes [18,19]. Recently, commercially produced raw bioplastics were found to be anaerobically digestible into carboxylates [20]. It was shown that only powdered PHB (polyhydroxybutyrate) and PHBV (poly(hydroxy-butyrate-co-hydroxy-valerate) fermented into a mixture of carboxylates (carbon recovery in the range of 10 to 18%) and biogas (carbon recovery in the range of 10% to 35%). This mesophilic fermentation took 56 days but a significant part of the materials remained undegraded. PLA and some other biodegradable plastics did not lead to acidogenic fermentation, i.e., formation of microbially produced carboxylates. Another study from our group first hydrolyzed PHBV and consequently, with apparent 100% carbon efficiency, fermented the hydrolyzed part with mixed-cultures into acetate and n-butyrate. PLA is currently one of the most popular biodegradable plastics used, for example, for food packaging. So far, fermentation of solid PLA or hydrolyzed PLA into carboxylates like VFAs (e.g., acetic acid) has not been reported.

Several PLA-based products have the ‘compost OK label’ which directs that they can be deposited within organic waste streams for composting [7]. It is known that some types of PLA are digestible into biogas [21]; however, the actual microbial bioprocesses and intermediates were not reported. Using a two-stage process, it was shown that PLA is hydrothermally convertible into lactic acid, an α-hydroxy carboxylic acid, and consequently anaerobically digestible into biogas [22]. Here, it was theorized that during biogas production PLA is first hydrolyzed into lactic acid/lactate and consequently fermented to propionate and/or acetate by well-known bioprocesses; these biochemicals can consequently be converted into biogas via other bioprocesses [9]. We hypothesize that such lactic acid derived from PLA can also be fermented into other carboxylates like acetate and n-buty-rate by various known bioprocesses [9]. Therefore, short-chain carboxylates could be chain-elongated using the remaining lactate up to medium-chain carboxylates once the various bioprocesses are combined within a bioreactor [11,23].

The aim of this work was to assess the proof of concept of converting real PLA packaging materials with an industrial compostable label into carboxylates via a hydrolysis and consequent mixed-culture fermentation process. To do so, PLA food packaging waste (PLA-FPW) was collected, partly hydrolyzed at 70 °C for 21 days, whereafter the hydrolyzed materials were collected, and next fermented from day 21 until day 58 by a mixed culture. The microbial inoculum used in this study was obtained from a mixed-culture process which produced a spectrum of carboxylates from food waste, wherein lactate served as an intermediate [24]. Throughout this paper, biochemicals are both referred to as their acid as well as their salt form; this represents the total amount of both species unless otherwise stated.

## 2. Materials and Methods

### 2.1. Materials and Preparation

**Figure 1:**
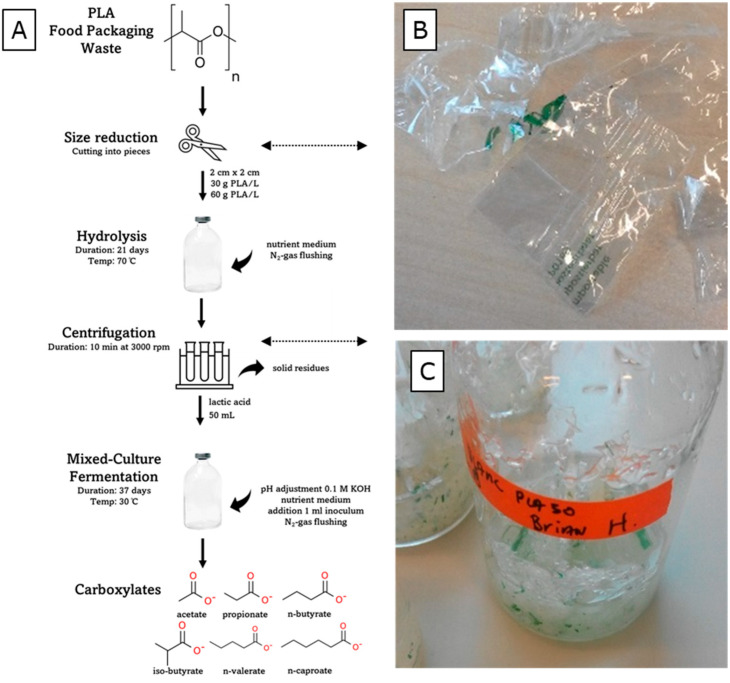
(**A**) Illustration of materials and experimental process. (**B**) Partly cut PLA food packaging waste. (**C**) Reactor with remaining non-hydrolyzed PLA-FPW solids after 21 days of hydrolysis.

The materials and experimental process used in this study are illustrated in Figure 1A. PLA food packaging waste (PLA-FPW) was obtained from vegetables packed in PLA-based wrapping that was purchased from the local Jumbo supermarket in Wageningen, the Netherlands. The material made a crackling sound when bent. The PLA-FPW material was certified industrially compostable according to the ‘Seedling label’ with the identifier ‘7P0189′ printed with green ink [7].

The film material was cut into 2 by 2 cm pieces using scissors (see Figure 1B). The paper-based label on the packaging material was not used. The water-rich medium (pH 7) used for the hydrolysis was a microbial growth medium (without inoculated micro-organisms) that was used previously in an anaerobic digestion study [25]. The macronutrient stock solution used (6 mL/L) contained KH_2_PO_4_ (37 g/L), CaCl_2_·2H_2_O (8 g/L), MgSO_4_·7H_2_O (9 g/L), yeast extract (1g/L), and NH_4_Cl (170 g/L). The trace element stock solution used (0.6 mL/L) contained FeCl_3_·4H_2_O (2 g/L), CoCl_2_·6H_2_O (2 g/L), MnCl_2_·4H_2_O (0.5 g/L), CuCl_2_·2H_2_O (30 mg/L), ZnCl_2_ (50 mg/L), HBO_3_ (50 mg/L), (NH_4_)_6_Mo_7_O_24_·4H_2_O (90 mg/L), Na_2_SeO_3_·5H_2_O (100 mg/L), NiCl_2_·6H_2_O (50 mg/L), and EDTA (1 g/L). The mixed-microbial-culture inoculum used for the fermentation process was obtained from a lab bioreactor for short-chain fatty acid production as well as lactate microbial chain elongation processes from food wastes [24]. The inoculum was retrieved from 50 mL of effluent, which was centrifuged at 4000 rpm for 10 min. A pellet was formed in the centrifugation tube and the supernatant was removed. In this way, the remaining lactate and other fermentation products present in the effluent broth were removed. The pellet was suspended in 20 mL of demi water and was used as the inoculum. A 1 mL volume of inoculum per bioreactor serum flask was used. The inoculum was a mixed culture, as analyzed by the previous study, containing various species including from the genera *Caproiciproducens*, *Lactobacillus*, and *Clostridium sensu stricto 12* [24].

### 2.2. Experimental Procedures

The experiments were conducted in triplicate and the blanks in duplicate. First, pretreatment hydrolysis was started by adding 1.5 or 3.0 g of PLA-FPW and 50 mL of microbial growth medium into a serum flask reactors with a total working volume of 250 mL (including headspace). The reactors containing 30 or 60 g/L PLA-FPW were flushed for 30 s with N_2_ gas, closed with a rubber stopper with aluminum cap, and placed in a 70 °C temperature-controlled shaker at 100 rpm. The blank reactors without PLA-FPW were prepared similarly while omitting the addition of the PLA-FPW materials. The reactors were opened after 21 days of operation. The pretreatment hydrolysis experiments were followed by the fermentation experiments. The hydrolyzed plastic containing medium and lactic acid was obtained by separating the remaining PLA solids through decanting the medium into a centrifuge tube and centrifuging the medium at 3000 rpm for 10 min. For the mixed-culture fermentation, 40 mL of the hydrolyzed plastic-containing medium was used; 1 mL of prepared inoculum was added as well as 10 mL of fresh medium, and the pH was adjusted to 5.3, 5.4, and 6.6 using 5.6 to 6.7 mL of 0.1 M KOH for the ‘30 g/L PLA-FPW’ experiment. For the ‘60 g/L PLA-FPW’ experiment, the pH was adjusted to 5.3, 5.4, and 6.5 using 11.3 to 11.6 mL of 0.1 M KOH. Serum flasks with a total working volume of 250 mL (including headspace) were used as bioreactors. The bioreactors initially contained, on average, 8.7 (±1.5) g/L lactate for the ‘30 g/L PLA-FPW’ experiment and 15.5 (±1.6) g/L lactate for the ‘60 g/L PLA-FPW’ experiment. Eventually, the headspace of the bioreactors were flushed for 30 s with N_2_ gas, closed with a rubber stopper with aluminum cap, and placed in a 30 °C temperature-controlled shaker at 100 rpm. The fermentation continued from day 21 until day 58. The pH was not adjusted during fermentation.

### 2.3. Analytical Methods

A 1.5 mL volume of the liquid samples was regularly taken using a syringe pierced through the rubber stopper. Before analysis, the samples were centrifuged and filtered with a 0.45 µm membrane (CHROMAFIL Xtra, Machinerey-Nagel, Düren, Germany) to remove solid residues like non-hydrolyzed plastics and micro-organisms. During hydrolysis of the PLA-FPW, a total of 10.5 mL of sample was taken from the total volume of 50 mL of microbial medium. These samples may have contained only small plastic particles that could go through the syringe. This means that, during the hydrolysis experiment, the total liquid medium volume decreased and that the actual formed lactic acid concentration, in the case where no samples were taken, would have been lower. It was decided to present the actual measured concentrations. During the mixed-culture fermentation, liquid samples were also taken, similar to the process during the hydrolysis experiment. From most samples, pH was measured during hydrolysis and fermentation by an off-line pH probe (Mettler Toledo, Tiel, The Netherlands). L-lactate was measured during hydrolysis and fermentation by high-performance liquid chromatography (HPLC) using a previously published protocol [24]. The PLA hydrolysis efficiency was calculated based on the supplied PLA and amount of lactic acid produced, taking into account the lactic acid removed due to sampling and the reduced liquid volume at the end of the hydrolysis experiments. The HPLC instrument (Thermo Scientific Dionex UltiMate 3000, Thermo Fischer, Karlsruhe, Germany) was equipped with a refractive index detector (Shodex RI-71, Separations) and an Alltech OA-1000 column (length 300 mm; ID 6.5 mm). The column oven was maintained at 60 °C and 1.25 mM sulfuric acid was used as mobile phase at a continuous flow of 0.6 mL/min. The injection volume was 20 μL. The chromatography data were analyzed with Chromeleon software (version 6.80 SR13; Thermo Fischer, Karlsruhe, Germany). In the samples taken during fermentation, other carboxylic acids, i.e., straight-chain fatty acids and alcohols (with carbon chain lengths of C2–C8), iso-butyrate, b-valerate (both 2- and 3-methylbutanoic acids together), and iso-caproate (4-methyl-pentanoic acid), were also measured by gas chromatography (GC, Agilent, Wilmington, NC, USA) methods using a previously published protocol [26]. The carboxylate recovery from the provided lactate was calculated, taking into account the amount of carboxylates produced in the removed samples and the amount of carboxylates at the end of the fermentation experiment. 

## 3. Results

### 3.1. PLA Food Packaging Waste Was Hydrolyzed into Lactic Acid within a Microbial Growth Medium

**Figure 2:**
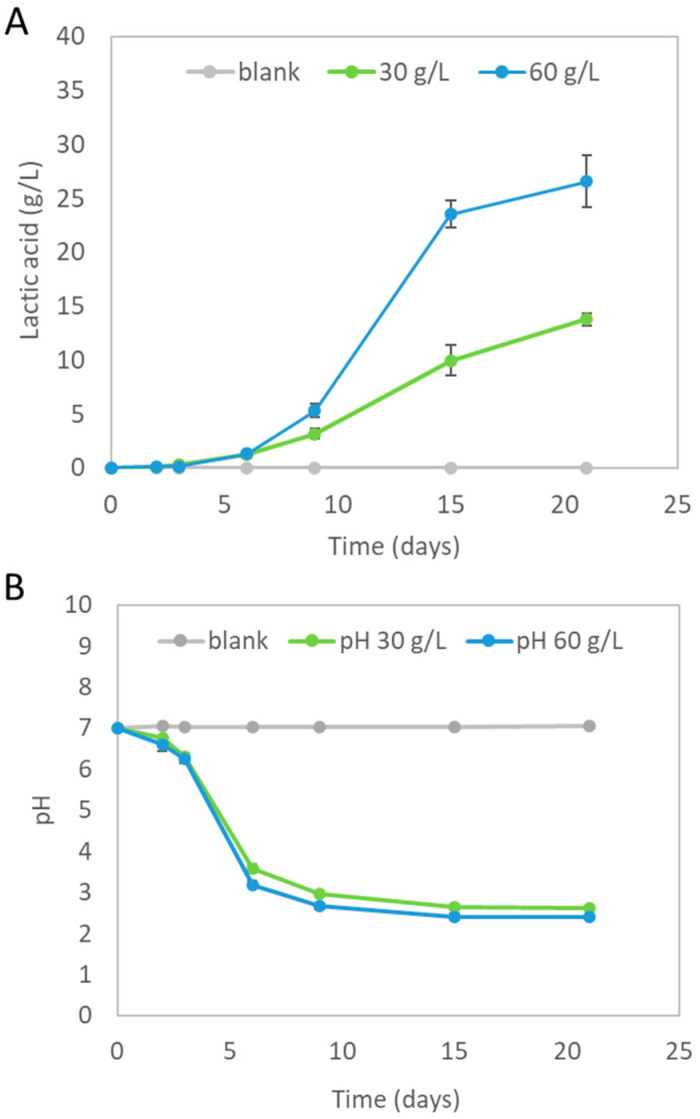
(**A**) Average lactic acid concentration measured at each sampling time during PLA-FPW hydrolysis (at 0 = blank and 30, 60 g/L PLA-FPW added) in microbial growth medium at 70 °C. (**B**) pH profile during PLA-FWP hydrolysis. Error bars (not always visible) represent standard deviation.

An end-of-life bioplastic food packaging was successfully hydrolyzed within a complex microbial growth medium containing nutrients at 70 °C. Lactic acid formation occurred during PLA-FPW hydrolysis. From the experiments ‘30 and 60 g/L PLA-FPW’, an average lactic acid concentration of, respectively, 13.8 (±0.60) and 26.6 (±2.42) g/L was measured at the end of the experiment (see Figure 2A). This corresponded to an average PLA hydrolysis efficiency of 37.8 to 39.1%. The starting PLA-FPW concentrations were chosen to be able to reach lactate concentrations higher than 10 g/L, which was previously shown to be suitable for the production of carboxylates via mixed-culture fermentation [23]. The double amount of initial PLA-FPW resulted in an almost double (1.9 times) amount of lactic acid produced. The highest volumetric lactic acid formation rates (1.1 and 3.0 g/L×d) occurred from day 9 to day 15 for the ‘30 and 60 g/L PLA-FPW’ experiments, respectively. No lactic acid was detected in the blanks. Evidently, the higher the amount of PLA-FPW added, the higher the volumetric hydrolysis rate was, and eventually this led to the highest measured lactic acid concentration.

Not all of the supplied PLA-FPW material was hydrolyzed; by eye, the 2 by 2 cm parts were visibly getting smaller and the solid plastic material which contained the green ink of the ‘Seedling label’ remained (see Figure 1B). During the ‘30 and 60 g/L PLA-FPW’ hydrolysis experiment, the pH decreased from 7.0 to, respectively, 2.40 (±0.04) and 2.62 (±0.01) (see Figure 2B). The pH buffer capacity, which is part of microbial growth media, did not suffice to prevent dropping out of neutral pH conditions. The pH of the blank experiment remained neutral around pH 7.

### 3.2. Lactate Obtained from PLA Food Packaging Waste Was Fermented into a Spectrum of Carboxylates

**Figure 3:**
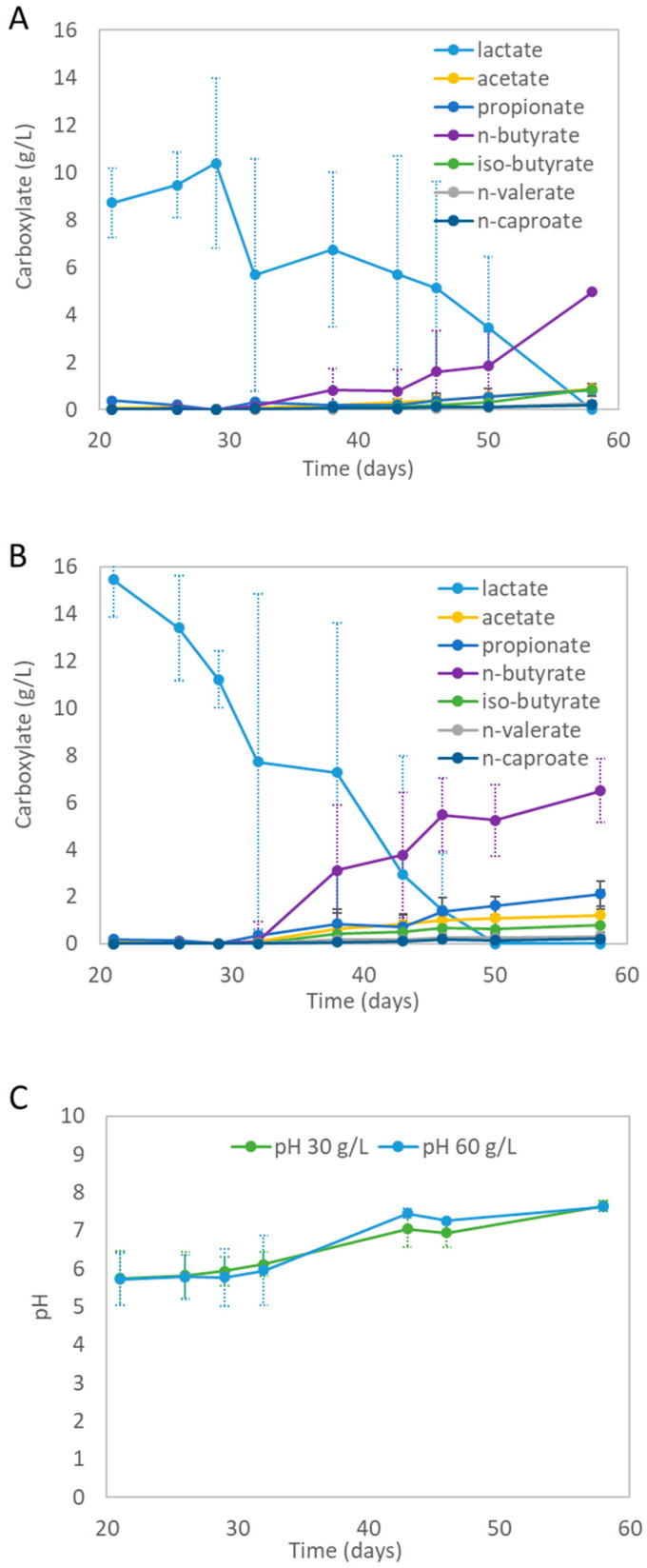
(**A**) Average carboxylate concentration measured during mixed-culture fermentation of lactate obtained from hydrolyzed PLA-FPW for the ‘30 g/L PLA-FPW’ experiment. (**B**) Average carboxylates concentration measured during mixed-culture fermentation of lactate obtained from hydrolyzed PLA-FPW for the ‘60 g/L PLA-FPW’ experiment. (**C**) pH profiles during fermentation. Error bars (not always visible) represent standard deviation.

The mixed-culture fermentation experiments converted the provided lactate into a spectrum of carboxylates (see Figure 3A,B). On day 38 (after 17 days of fermentation), the first new carboxylates were formed during all experiments.

The average lactate concentration dropped with the formation of various biochemicals. Both experiments produced the same kinds of carboxylates. The highest final average concentrations were achieved with the ‘60 g/L PLA-FPW’ experiment, for which the average concentrations were as follows: 1.2 ± 0.5 g/L acetate (C2), 2.1 ± 1.0 g/L propionate (C3), 6.5 ± 1.4 g/L n-butyrate (C4), 0.8 ± 0.7 g/L iso-butyrate (i-C4), 0.3 ± 0.2 g/L n-valerate (C5), and 0.2 ± 0.2 g/L n-caproate (C6); see Figure 3A. On average, n-butyrate was the main identified product at 6.5 g/L. Alcohols, heptanoate (n-C7), and octanoate (n-C8) were not detected. The blank experiments did not show significant biochemical formation (see Supplementary Materials, Figure S1A,B). During fermentation, lactate was apparently completely consumed, which coincided with the formation of other carboxylates. With the new carboxylates, 75 to 92% of the supplied carbon from the lactic acid was recovered from the ‘30 g/L’ and ‘60 g/L PLA-FPW’ experiments, respectively. This shows that the carboxylates were the key products. The visible increase in turbidity was evidence for the growth of micro-organisms. The possible presence of plastic additives, which may have leached during the hydrolysis of the PLA-FWP, did not hinder the production of carboxylates. The pH was not controlled during the mixed-culture fermentation and on average increased from 5.8 (±0.7) to 7.7 (±0.1) for the ‘30 g/L PLA-FPW’ experiment and from 5.7 (±0.7) to 7.6 (±0.02) for the ‘60 g/L PLA-FPW’ experiment (see Figure 3C). The pH of the blank experiments hardly changed (see Figure S1C in Supplementary Materials).

## 4. Discussion

### 4.1. Prospects for Microbial Recycling of End-of-Life PLA-Based Products

This work shows the feasibility of microbially recycling a real PLA packaging material, with an industrial compostable label, into carboxylates. This is a key step to unlock PLA-based end-of-life products as feedstock for the carboxylate platform. In the present study, a clean PLA-FPW material was used. If a real PLA-post-consumer waste is obtained, other materials like other plastics or contaminations could be present depending on the actual case. The produced carboxylates, like n-butyrate, could, in theory, be useful in producing new biodegradable plastic products by microbial polyhydroxyalkanoate (PHA) formation [27]. Carboxylates could also be utilized for various other applications like nail polishers, lubricants, plasticizers, etc. [28]. To recover BDPs like PLA, of course, one also should take into account the quality of the produced carboxylates. BDPs can contain various additives [29]. It is not simply known which additives are used in a certain BDP product and what happens with them during mixed-culture fermentation. Several additives used in (bio)plastics do, for example, inhibit the methanogenesis process in anaerobic digestion [30]. Various BDPs, as well as non-biodegradable plastics, do end up in the organic fraction of municipal solid waste [31]. Which precise plastic additives are used and what really happens with these in full-scale anaerobic digesters is not completely known [30,32]. Knowledge about this, as well as knowledge on hazards and human exposure, and the ecotoxicity of plastics, their additives, and degradation products is needed for risk-based assessments [33,34].

PLA is the most produced biodegradable biobased plastic polymer and the real-life examples of its chemical recycling applications are ongoing [35,36,37]. Other recycling routes that are mentioned to be potentially feasible include mechanical recycling as well as organic recycling via composting [38]. In the latter process, in theory, CO_2_ is taken up by the plant to make biomass which is processed into PLA, which is then, during composting, released into the atmosphere to close the cycle. To what extent these various recycling routes are actually happening is, to the best of the authors’ knowledge, not documented. With the wide variety of PLA-based products, it is plausible that various end-of-life routes will be developed for biodegradable plastics as is currently happening for non-biodegradable plastics [39,40,41]. Beside packaging materials, PLA is also used in various other products; for example, in bean bags as a biofoam [42]. Moreover, PLA is also used in composites, for example, in 3D printing filaments consisting of PLA and PHA [43]. Three-dimensional printing can be applied to produce various products like chairs and other attributes [44]. How to effectively recycle all these products at the end of their life is still an open question. Waste management systems are under development. For example, it is known that PLA materials can be recovered from mixed plastic waste streams [38]. With the potential increased use of PLA materials, up, down, or recycling processes of these materials may appear. Here, microbial recycling processes could play a role; for example, by potential co-fermentation of hydrolyzed PLA, as we have demonstrated here, from the organic fraction of municipal solid waste which also can be converted into carboxylates [10]. The potential advantages of this co-digestion are that microbial nutrients are present in the organic wastes and do not have to be added in the case of PLA end-of-life products without nutrients. Secondly, a small fraction of PLA can already contribute to the production of carboxylates. With the further expected increase in PLA production [35], as well as more PLA waste generation, the ability to recycle PLA-based end-of-life products is becoming more significant. Therefore, the microbial recycling process of PLA into carboxylates proposed here has potential to be realized.

### 4.2. Improving Hydrolysis and Development of PLA and other BDP Converting Bioprocesses

The hydrolysis of PLA polymers is widely studied. The experimental results presented here showed that not all the PLA-FPW materials were hydrolyzed into lactic acid. However, since the lactic acid concentration was still increasing at the last measured datapoint (see Figure 2A), a longer reaction time could be required to further hydrolyze the PLA. Some of the provided PLAs could be present as oligomers, which are expected to be present in a concentrated solution of lactic acid [23]. These may have been further hydrolyzed into lactate during the actual fermentation process and served as new substrate for the bioprocess. One observation was that some plastic particles with green ink from the ‘Seedling label’ remained. What happens to this part of the PLA-FPW during long-term hydrolysis (and consequent fermentation) will be interesting to study. Other parameters which could be studied for this specific material are the temperature and pH, since these are mentioned as the main factors influencing hydrolysis [45,46]. pH impacts on PLA hydrolysis both via affecting the reaction mechanism and kinetics [45]. In this study, during the hydrolysis experiment, the pH dropped from neutral to more acidic conditions. The PLA hydrolysis took place within a microbial growth medium with a buffering capacity which was not sufficient to maintain the more neutral pH starting conditions. In the case where one would like to develop a bioprocess whereby PLA hydrolysis and microbial fermentation to carboxylates should co-occur, pH control is advised. These fermentation processes do typically occur around pHs 5 to 7, which is evidently higher than the average pHs of 2.40 (±0.04) and 2.62 (±0.01) reached at the end of the hydrolysis experiment [11,23,24,47,48,49]. In such acidic media, hydrolysis of the PLA ester bonds is catalyzed by protons [45]. pH control can therefore be considered as relevant to develop an efficient hydrolysis process. Moreover, it is also relevant to know the PLA’s material properties, since they can also influence hydrolysis and could give further insights into how to improve the depolymerization process. The addition of enzymes or micro-organisms could also be beneficial since they can play a role in the actual hydrolysis process [50]. However, a recent study showed that ‘new’ PLA milled bioplastic beads were not fermentable under mesophilic conditions [20]. Alternatively, PLA may be easier to ferment into carboxylates under thermophilic conditions; it has already been shown that PLA is digested faster into biogas at higher temperatures [51]. The present research showed, for the first time, that PLA is fermentable into carboxylates with the formation of a spectrum of C2–C6 multi-carbon carboxylates.

The observed broad carboxylate product spectrum from C2 to C6 was potentially due to the occurrence of various microbial bioprocesses. Contreras and co-workers provided a comprehensive overview of the thermodynamically feasible bioprocesses and proposed reactions for lactate-based mixed-culture fermentation (see equations 1 to 4, a selection from [23]).

3 lactate^−^ → 2 propionate^−^ + acetate^−^ + CO_2_ + H_2_O ∆G0′ = −115.7 kJ/reaction (1)

lactate^−^ + acetate^−^ + H^+^ → n-butyrate^−^ + CO_2_ + H_2_O ∆G0′ = −57.9 kJ/reaction (2)

lactate^−^ + propionate^−^ + H^+^ → n-valerate^−^ + CO_2_ + H_2_O ∆G0′ = −57.8 kJ/reaction (3)

lactate^−^ + n-butyrate^−^ + H^+^ → n-caproate^−^ + CO_2_ + H_2_O ∆G0′ = −57.9 kJ/reaction (4)

For example, lactate could be converted into both acetate and propionate (Equation (1)). These biochemicals can be chain-elongated to n-butyrate and consequently to caproate or n-valerate, respectively (Equations (2)–(4)). Also, iso-butyrate formation was observed during the experiments. Iso-butyrate formation during mixed-culture culture chain elongation processes was discovered during methanol-based chain elongation processes [52]. Iso-butyrate formation was also observed by isolated microorganisms obtained from mixed-culture lactate chain-elongating organisms. These organisms produce acetate, n-butyrate, iso-butyrate, and n-caproate [53]. Iso-butyrate does not always appear in mixed-culture lactate chain elongation processes. A study which used sunflower oil during an extractive fermentation process showed that iso-butyrate was especially feasible during the extraction phase at both pH 5.0 and 7.0 [48]. In the present study, pH was not actively controlled during fermentation and steadily increased. Such pH variation could support the metabolic activity of various microbial species, since this parameter is known to be selective for mixed-culture processes [9,54]. Lactate-based chain elongation processes were originally reported to be favored in mildly acidic conditions. Under slightly higher pH conditions (>6), lactate may undergo other fermentation pathways that lead to propionate production as discussed earlier [55]. Interestingly, pH dynamics during organic waste primary fermentation processes combined with lactate-based chain elongation processes can also be exploited by fed-batch operation [24]. During primary fermentation, lactate, as well as short-chain fatty acids like acetate, are produced with a consequent drop in pH. During lactate-based chain elongation, the pH increases again. Both processes are stimulated by fed-batch operations, which will likely reduce the need for pH control compared to processes where both steps have been separated [24]. In a similar way, hydrolyzed PLA (at a low pH) could be used in a continuous lactate-based chain elongation process to support pH control and reduce the need for acid to lower the pH during lactate-based chain elongation. Future studies with continuously operated bioreactors at different controlled pH levels can provide more insight into the role of pH, the formation of specific carboxylates, and the role of specific microorganisms in the different bioprocess reactions.

Various BDP waste materials may be mixed together and used to produce carboxylates. Moreover, it was also recently shown that PHA could be first hydrolyzed under alkaline conditions and consequently fermented by mixed-culture micro-organisms into acetate and n-butyrate [17]. This anaerobic fermentation process occurred under quite similar conditions within a pH of about 7.0 to 6.1. Therefore, both PLA and PHA end-of-life products (or even composites of them like 3D-printed objects [43]) could be co-fermented all into multi-carbon carboxylates. Moreover, other BDPs like starch-based plastics are known to be anaerobically digestible and are hypothesized to be convertible to carboxylates [17]. This all indicates that BDPs could become a potential feedstock for various microbial recycling processes.

## 5. Conclusions

This study demonstrated a proof of concept for a microbial recycling process consisting of PLA food packaging waste conversion into C2–C6 multi-carbon carboxylates via hydrolysis and mixed-culture fermentation. To do so, the bioplastic was first cut into pieces and hydrolyzed ~40% within a microbial growth medium. It was shown that a higher concentration of added bioplastic waste leads to a higher concentration of lactic acid being reached more quickly. The obtained lactate was consequently fermented into a spectrum of short- and medium-chain carboxylates with an efficiency of up to ~90%. n-butyrate was the most dominant product, with an average concentration of 6.5 g/L. Therefore, end-of-life PLA-based materials could become a useful substrate for the carboxylate platform and contribute to the circular economy.

## Data Availability

The original data presented in this paper will be available at the 4TU Research Database via this site: https://data.4tu.nl/articles/dataset/3011851a-6248-44ce-8a41-8d407bc9d3d0 (accessed on 17 August 2023).

## Supplementary Materials

The following supporting information can be downloaded at: https://www.mdpi.com/article/10.3390/microorganisms11082103/s1. Figure S1A: Carboxylate concentrations measured in blank experiment 1; Figure S1B: Carboxylate concentrations measured in blank experiment 2; Figure S1C: pH profile of blank experiment during fermentation.
