# Supplementary material for: Microbial Recycling of Polylactic Acid Food Packaging Waste into Carboxylates via Hydrolysis and Mixed-Culture Fermentation
Source: Microorganisms. 2023 Aug 18;11(8):2103. doi: 10.3390/microorganisms11082103 (PMC10458239; doi:10.3390/microorganisms11082103)
Supplement: Supplementary file 1 [file microorganisms-11-02103-s001.zip › microorganisms-2453639-supplementary.pdf]

microorganisms

MDPI

Supplementary Materials

Microbial Recycling of Polylactic Acid Food Packaging Waste into Carboxylates via Hydrolysis and Mixed-Culture Fermentation

David P.B.T.B. Strik[1]*, Brian Heusschen[1]

[1] Environmental Technology, Wageningen University & Research, 6708 WG Wageningen, The Netherlands
* Correspondence: david.strik@wur.nl

Blank figures

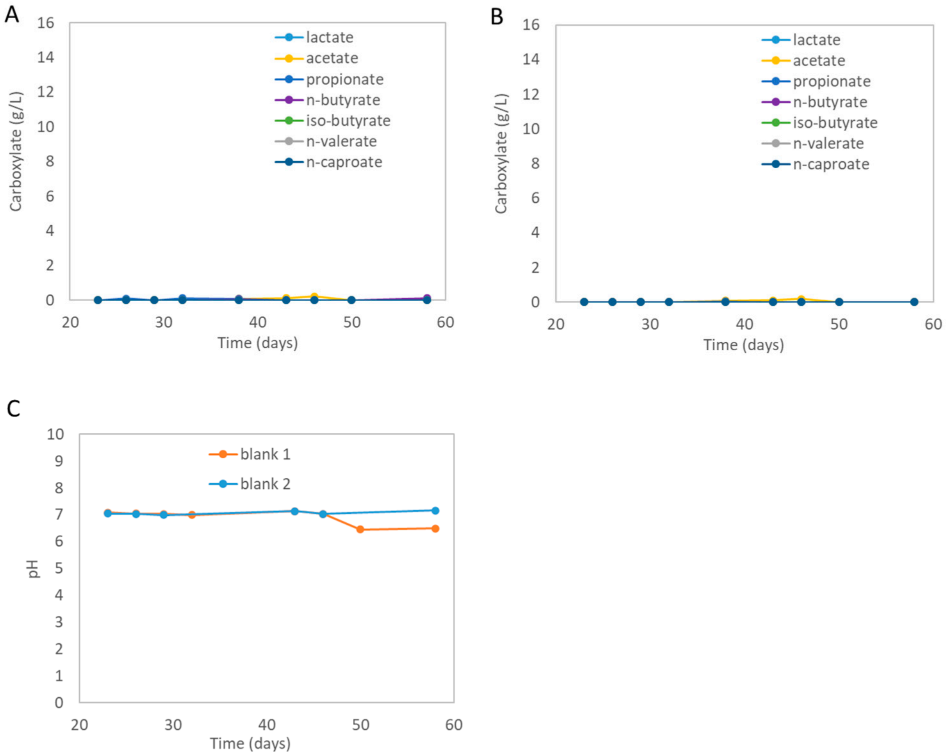

Figure S1.A. Carboxylates concentration measured of blank experiment 1; **B.** Carboxylates concentration measured of blank experiment 2; **C.** pH profile of blank experiment during fermentation.
